# Protective Factors Buffer Life Stress and Behavioral Health Outcomes among High-Risk Youth

**DOI:** 10.1007/s10802-019-00515-8

**Published:** 2019-02-22

**Authors:** Shubam Sharma, Brian Mustanski, Danielle Dick, John Bolland, Darlene A. Kertes

**Affiliations:** 1 Department of Psychology, University of Florida, Gainesville, FL 32611-2250 USA; 2 Feinberg School of Medicine, Northwestern University, Evanston, IL USA; 3 Department of Psychology, Virginia Commonwealth University, Richmond, VA USA; 4 Department of Psychology, University of Alabama, Tuscaloosa, AL USA

**Keywords:** Adolescence, Behavioral health, Protective factors, Life stress, Internalizing problems, Externalizing problems, Substance use, Parenting, Neighborhood factors, High-risk environments

## Abstract

This study investigated internalizing problems, externalizing problems, and polydrug use among African-American youth residing in high-poverty neighborhoods, and tested the potential protective effects of religiosity, parental monitoring, and neighborhood collective efficacy on life stress and behavioral health outcomes (*N* = 576; 307 females; Mage = 16 years, SD = 1.44 years). A cumulative risk index reflected the combined effects of past year exposure to stressful life events, racial discrimination, and exposure to violence along with poor neighborhood ecology. Structural equation modeling revealed that cumulative risk significantly predicted internalizing problems, externalizing problems, and polydrug use. Interaction tests showed that the association of cumulative risk with internalizing problems was buffered by adolescent religiosity and neighborhood collective efficacy. The association of cumulative risk with externalizing problems was buffered by parental monitoring and collective efficacy. Adolescent sex further moderated these effects. The findings of the present study collectively highlight potential for protective factors to buffer effects of cumulative risk on behavioral health outcomes among youth residing in high-risk neighborhoods.

## Introduction

Adolescence is a highly dynamic and vulnerable life phase throughout which behavioral health outcomes, such as internalizing problems, externalizing problems, and drug use may be exacerbated due to exposure to life stressors. Integrative models of developmental competencies among minority children highlight how the interaction of social position, racism, and segregation constructs adverse conditions and increases risk for poor adjustment outcomes, sometimes in gender-specific ways (Garcia-Coll et al. 1996). For youth in high poverty neighborhoods, this may include exposure to violence and neighborhood problems. African American youth, who disproportionately comprise youth in urban neighborhoods of concentrated poverty, also contend with the additional stress of racial discrimination.

In real-world settings and especially high-risk settings, stressors rarely occur in isolation. Developmental risk models posit that cumulative risk represents the additive effect of multiple interrelated risk factors an individual may encounter across individual, family, and environmental contexts (Evans et al. 2013). According to this view, exposure to multiple risk factors taxes one’s ability to cope and augments the risk for poor developmental outcomes (Evans et al. 2013). The cumulative risk approach examines the number of risks an individual encounters, rather than the intensity or pattern of the exposure to risk, and is operationalized by summing across multiple different risk factors (Evans et al. 2013). Cumulative risk, when operationalized with at least three or more risk factors, has been associated with conduct problems and depressed mood (Gerard and Buehler 2004), poor school achievement (Gassman-Pines and Yoshikawa 2006), and tobacco, alcohol, and other drug use (Adelmann 2005). Thus, examining the effects of high levels of risk factor exposure on adolescent behavioral health outcomes maximizes generalizability of research results to the growing number of children in America who confront multiple stressors.

Despite stressors co-existing in areas of concentrated poverty, protective factors may buffer the negative impacts of stressors (Zimmerman et al. 2013). Investigating protective factors from multiple levels of the social environment, including intrapersonal, interpersonal, and neighborhood levels, allows for more thorough analysis of factors influencing developmental outcomes (Bronfenbrenner 1994). Among the protective factors implicated in adolescent behavioral health outcomes are religiosity, parental monitoring, and neighborhood collective efficacy. Religiosity and collective efficacy, specifically, may provide opportunities for positive interactions with mentors that may influence the impact of cumulative risk on behavioral health outcomes (Cohen and Wills 1985; Mosavel et al. 2015). Parental monitoring has been also shown to be a salient protective factor, especially for African American adolescents (Lac and Crano 2009). This study investigates how these protective factors moderate associations of cumulative risk and behavioral health outcomes among African American adolescents residing in high-poverty neighborhoods.

### Adolescent Behavioral Health Outcomes and Stress

This study evaluates three behavioral health outcomes, internalizing problems, externalizing problems, and polydrug use, which exhibit negative short-term impacts as well as long-term risk for more severe outcomes in adulthood (Kelly et al. 2015). Internalizing problems consist of depressive, anxious, and withdrawn behaviors and somatic complaints; externalizing problems consist of conduct problems and aggressive behaviors (Achenbach and Rescorla 2001). Polydrug use, a common framework to test illicit substance use, refers to alcohol, tobacco, and marijuana use among adolescents (Kelly et al. 2015). Substance use among adolescents typically begins by experimentation with alcohol, tobacco, and marijuana before progressing to harder drug use, abuse, and addiction (Huang et al. 2011). Internalizing and externalizing problems are highly comorbid, which is indicative of shared risk and protective factors (Liu et al. 2016). Polydrug use is comorbid with mood problems and delinquency (Huang et al. 2011; Salom et al. 2016). Adding to the complexity, sex differences have been reported for internalizing and externalizing problems (Moilanen et al. 2010; Liu et al. 2016; Rai et al. 2003). Moreover, polydrug use has been shown to increase throughout adolescence, with greater linear change across adolescence among males (Elkington et al. 2011).

Stressors in high-poverty environments, such as major life events, violence, racial discrimination, and poor neighborhood ecology, are documented risk factors for the development of internalizing problems, externalizing problems, and polydrug use. Violence exposure and racial discrimination can exacerbate internalizing (Mustanski et al. 2013; Gorman-Smith and Tolan 1998) and externalizing problems among African American adolescents (Deane et al. 2016). Poor neighborhood ecology is characterized by social disorder and crime (e.g., theft, street drugs), physical dishevel of the neighborhood (e.g., vandalism, litter, vacant housing), and residential instability. Neighborhood ecology is associated with substance use and internalizing problems (Assari et al. 2015). Because these stressors often co-exist in high-poverty environments, it is necessary to expand beyond single-variable analysis of risk to examine the impacts of cumulative risk on minority adolescents’ development.

### Protective Factors

A protective factor is conceptually distinct from a risk factor (Rutter 2000). In the traditional risk-outcome approach, emphasis is placed on identifying and reducing risks, and protection is often operationalized as absence of risk (i.e., high neighborhood violence as risky and low neighborhood violence as protective; Zimmerman 2013). In contrast, a strength-oriented approach considers protective factors that are distinct from risk factors (Rutter 2000). This study examines three protective factors that reflect a continuum from the intrapersonal to neighborhood level in terms of their direct associations with behavioral health outcomes and moderating role on stress-outcome associations. Protective influences are typically termed “protective” when they moderate negative effects of risk for predicting behavioral health outcomes, but termed “promotive” when they directly associate with outcomes (Zimmerman et al. 2013). Acknowledging that a positive environmental factor can exhibit both promotive and protective effects, for simplicity we hereafter refer to these variables as protective factors. Protective factors may moderate associations of cumulative risk and behavioral health outcomes by offering adolescents opportunities for personal development (Mosavel et al. 2015). The proposed protective factors may grant opportunities for community involvement, establishing relationships with and oversight by mentors, internalization of parental or community values, and practicing social skills, all of which may contribute to positive youth development.

#### Religiosity

Religiosity is a documented protective factor for substance use and delinquency among adolescents (Berg et al. 2009). Religiosity, which is conceptually distinct from spirituality (Reutter and Bigatti 2014), is often assessed as religious involvement (i.e., participation in religious activities) and importance of religion to the self. Religious involvement is associated with lower internalizing (Mattis and Mattis 2011) and externalizing problems (Salas-Wright et al. 2013), and can serve as a positive avenue of socialization, providing adolescents with emotional and moral behavior support (Yonker et al. 2012). Religiosity is associated with higher self-esteem, optimism, and emotional regulation, and may protect against psychosocial problems related to experiencing stressors in high-poverty contexts (Lee and Neblett 2017).

#### Parental Monitoring

Parental monitoring, operationalized as the extent to which parents are aware of their children’s whereabouts and activities, influences adolescent decision-making and risky behaviors, and is especially protective in high-risk environments (Lac and Crano 2009). Low parental monitoring is associated with behavioral health problems, delinquency, peer deviance, pro-substance attitudes, and poor academic performance (Voisin et al. 2017; Lac and Crano 2009; Mann et al. 2015). Conversely, high parental monitoring can reduce internalizing and externalizing problems (Hamza and Willoughby 2011), and is associated with lower youth violence rates in high-risk neighborhoods (Fergus and Zimmerman 2005). Effective parental monitoring may indicate a high-quality parent-child relationship characterized by an adolescent’s disclosure to parents regarding their whereabouts, peer groups, and activities to parents (Kerr and Stattin 2000).

#### Collective Efficacy

Collective efficacy is defined as social cohesion and informal social control among individuals in a neighborhood (Sampson et al. 1997). High collective efficacy in a neighborhood is characterized by residents being socially connected, actively reinforcing rules for acceptable behavior, and readily intervening to maintain social control (Sampson et al. 1997). Low collective efficacy is linked with high rates of adolescent mental health problems and risky health behaviors (Voisin et al. 2011). High collective efficacy is associated with low rates of adolescent conduct problems and substance use (Widome et al. 2008). Because high collective efficacy represents strong social networks, including aspects of social interaction, community supervision, and established expectations that promote positive youth development (Morenoff et al. 2001), the degree of collective efficacy may moderate stress-outcome associations.

### Current Study

This study adds to the literature in multiple ways. First, this research adopts a cumulative risk approach capturing the additive effect of multiple sources of stress, which stands in contrast to the bulk of research examining environmental risk factors separately. Assessing cumulative risk is indicative of adolescents’ actual experiences of contending with many stressors in daily life, and thus exhibits high applicability to real-world high-risk environments (Zolkoski and Bullock 2012). Second, research on behavioral health outcomes has been extensively informed by the risk-outcome model approach, which seldom accounts for effects of protective factors (Luthar 2006; Masten 2001). To address this, this study highlights the role of protective factors from multiple ecological contexts of adolescents’ lives. This approach has greater feasibility for informing preventive intervention efforts, as successfully reducing stressors in high-risk settings remains difficult (Masten 2001). Third, adolescent perceptions of their neighborhoods, such as collective efficacy, are grossly understudied as a source of protective effects for critical youth outcomes in comparison to interpersonal factors such as parental monitoring (Zimmerman et al. 2013). Finally, previous studies that have examined sex differences in predictors of youth behavioral health outcomes have been limited in sample size, with data based on majority African American adolescents residing in high-poverty neighborhoods particularly scant. Thus, this study’s larger sample size allows a novel addition to the literature by assessing whether the buffering roles of the proposed protective factors vary by sex.

This research examines the proposed protective effects of religiosity, parental monitoring, and collective efficacy in association with cumulative risk and behavioral health outcomes within the context of a hard-to-reach population of predominantly African American adolescents residing in urban neighborhoods of concentrated poverty. Compared with their White peers, African American youth disproportionately reside in areas marked by economic instability and fewer school and community resources (Zolkoski and Bullock 2012). It was hypothesized that cumulative risk, an index of exposure to stressful life events, racial discrimination, exposure to violence, and poor neighborhood ecology, would be associated with higher levels of internalizing problems, externalizing problems, and polydrug use. With respect to protective factors, it was predicted that higher religiosity, parental monitoring, and collective efficacy would be associated with lower levels of internalizing problems, externalizing problems, and polydrug use, and that protective factors would also moderate the associations of cumulative risk and behavioral health outcomes, in effect buffering the negative effects of cumulative risk.

Specifically, based on previously documented direct associations of religious service participation and internalizing and externalizing problems (Mattis and Mattis 2011; Salas-Wright et al. 2013), it was hypothesized that higher religiosity would attenuate the associations of cumulative risk with internalizing and externalizing problems. Based on studies showing direct associations of parental monitoring and internalizing and externalizing problems, and substance use (Hamza and Willoughby 2011; Mann et al. 2015), it was predicted that parental monitoring would moderate the associations of cumulative risk with internalizing problems, externalizing problems, and polydrug use. Further, it was hypothesized that higher collective efficacy would moderate the associations of cumulative risk with externalizing problems and polydrug use, based on prior research showing direct associations of similar constructs (Widome et al. 2008). Based on previous reports of sex differences in behavioral problems, and consistent with the model of developmental competencies for minority children (Garcia-Coll et al. 1996), sex was also tested as a moderator of the proposed associations among cumulative risk, protective factors, and behavioral health outcomes (Rew and Wong 2006). Given the limited research related to sex differences in larger samples of African American youth residing in high-risk neighborhoods, tests of sex effects were considered exploratory.

## Method

### Participants and Procedure

This study was approved by the Institutional Review Boards of the University of Florida, University of Illinois at Chicago, Northwestern University, University of Alabama, Virginia Commonwealth University, and Washington University. Six hundred participants were recruited from low-income neighborhoods based on high poverty rates according to U.S. Census data ( 2012) in the greater metropolitan statistical area of Mobile, Alabama. Recruitment methods included using flyers and door-to-door visits by study staff (see Byck et al. 2013). Approximately 82% of participants resided in households with a yearly income of less than $20,000, and participants were demographically representative of the broader neighborhoods (see Bolland et al. 2016). Adolescents were aged 13–19 (*M*_*age*_ = 16 years, *SD* = 1.44 years, 307 females) with most self-identifying as African American (98.8% African American, 0.3% White, and 0.9% mixed race). Rates of missing data were low, with 576 (96%) of those initially recruited completing all assessments. Due to the hard to reach nature of the population, if more than one adolescent in a household expressed interest in participating, they were enrolled. Of the 576 total participants, 263 had a household member also in the study (*M* = 1.45 children per family, *SD* = 0.50). Statistical analyses were conducted adjusting for non-independent observations within households. Written consent was obtained from adolescents and their primary caregivers at the time of the study. Interviews were conducted at local community centers using interview-administered questionnaires and audio computer-assisted self-interview (ACASI) for privacy during assessment of sensitive subjects. All scales were self-reported by adolescents, who were compensated $30 for their participation.

### Measures

#### Cumulative Risk Factor

The following variables of environmental risk were used to generate the cumulative risk factor.

##### Neighborhood Ecology

The Neighborhood Ecology scale, adapted from the Neighborhood Problems scale (Gorman-Smith et al. 2000) was comprised of 14 items reflecting adolescents’ experiences of whether problems such as graffiti or drug paraphernalia were present or absent in their neighborhoods. Scores were summed, such that higher scores indicated more neighborhood problems. This scale has been shown to be internally consistent in previous samples of African American adolescents, with Cronbach’s alphas ranging from 0.82 to 0.83, and scores have been associated with measures of delinquency involvement (Gorman-Smith et al. 2000). Cronbach’s *α* in this sample was 0.83.

##### Exposure to Violence

The Exposure to Violence scale (Gorman-Smith and Tolan 1998) was comprised of nine items related to victimization and witnessing violence during the past 12 months, such as “Have you ever been robbed in your neighborhood during the past 12 months?” A total summary score was computed, with higher scores indicating more exposure to violence. This scale has demonstrated internal consistency in prior samples of African American adolescents, with Cronbach’s alphas ranging from 0.78 to 0.84, and scores have been associated with aggressive, depressive, and anxious symptomology (Gorman-Smith and Tolan 1998). Cronbach’s *α* in this sample was 0.80.

##### Exposure to Stressors

Exposure to Stressors, measured by the Stress Index (Attar et al. 1994; Gorman-Smith and Tolan 1998), was comprised of 16 items related to life transitions, circumscribed events, and violence exposure during the past 12 months, such as, “Did a family member die?” Scores were summed to form a total score of exposure to stressors, with higher scores indicating greater stress exposure. Internal consistency in previous studies with African American youth has ranged from alpha scores of 0.63 to 0.85, and scores have been associated with aggressive behaviors and internalizing problems (Gorman-Smith and Tolan 1998). Cronbach’s *α* in this sample was 0.78.

##### Racial Discrimination

Racial discrimination, assessed by the Schedule of Racist Events (Landrine and Klonoff 1996), was comprised of 14 items that examined participants’ exposure to racial discrimination during the past 12 months, such as how often they were treated unfairly by strangers because of their race. Responses were coded on a 0–2 scale, reflecting never, sometimes, or a lot for each item. The adolescent scale is an adapted version of the original 18-item Schedule of Racist Events used with adults. Scores were summed, with higher scores indicating more experiences of racist events. In samples of African American youth, this scale has shown high internal consistency, with alphas ranging from 0.94 to 0.96, and scores associated with perceived stress, lifetime history of disease, and alcohol use (Landrine and Klonoff 1996). In this sample, Cronbach’s *α* was 0.91.

#### Protective Factors

Protective factors included religiosity, parental monitoring, and collective efficacy. Guided by a social-ecological perspective, these were selected to reflect intrapersonal, interpersonal, and neighborhood-level factors, respectively.

##### Religiosity

Religiosity, examined by the Religiosity Scale (Landrine and Klonoff 1996), was comprised of two items assessing a) frequency of religious service attendance in the past year (never, less than once a month, once a month or more but less than once a week, and once a week or more) and b) importance of religion to the self (not important at all, fairly unimportant, fairly important, and very important). These two items were summed, with higher scores indicating higher religiosity. Because this scale only had two items, we followed the recommended practice of calculating the inter-item correlation as an assessment of scale reliability, with optimal Pearson’s correlation values ranging from 0.20–0.40 (Sijtsma 2009). The items that constituted the Religiosity Scale were moderately correlated (*r* = 0.44, *p* < 0.001).

##### Parental Monitoring

Parental monitoring, assessed by the Parenting Style questionnaire (Oregon Social Learning Center 1990; Donenberg et al. 2002), consisted of four Likert-style items, each with four possible response options, related to perceptions of the primary female caregiver. Example of items from this scale include, “How often do you let your caregiver know where you are going?” and “How often do you get to do things without telling your caregiver exactly where you are?” Scores were summed such that higher scores indicated more parental monitoring. In previous samples of African American youth, this scale has shown high internal consistency, with alphas ranging from 0.72 to 0.87, and higher scores have been associated with lower risky sexual behaviors and drug use (Donenberg et al. 2002). Cronbach’s *α* was 0.87 in this sample.

##### Neighborhood Collective Efficacy

Neighborhood collective efficacy, measured by the Collective Efficacy scale (Sampson et al. 1997; Morenoff et al. 2001), was comprised of nine items reflecting social cohesion, social control, and trust. Responses on this five-point Likert-type measure range from “very unlikely” to “very likely.” Scores were computed by taking the mean of responses. Scores were coded so that lower scores indicated lower collective efficacy, and higher scores indicated greater collective efficacy. In prior samples with African American adolescents, the Collective Efficacy scale has demonstrated reliability with alphas ranging from 0.79 to 0.88, and higher scores have been associated with lower rates of violence (Sampson et al. 1997). Cronbach’s *α* was 0.76 in this sample.

#### Behavioral Health Outcomes

##### Internalizing and Externalizing Problems

Internalizing and externalizing problems, assessed using the Youth Self Report (YSR; Achenbach 1991), was comprised of 112 items that measured psychosocial problems during adolescence. Internalizing problems was comprised of three subscales – withdrawn, somatic complaints, and anxiety and depression. Externalizing problems was comprised of two subscales – rule-breaking behavior and aggressive behavior. Raw scores were used in analyses, with higher scores on both internalizing and externalizing scales indicating greater internalizing and externalizing problems, respectively. The YSR has been previously shown to be a reliable and valid measure, with alphas ranging from 0.73 to 0.90, and scores associated with measures of life satisfaction and psychological functioning (Achenbach 1991). Cronbach’s *α* was 0.86 for internalizing problems and 0.89 for externalizing problems in this sample.

##### Polydrug Use

The AIDS Risk Behavior Assessment (ARBA; Watters 1994), a measure of health-risk behaviors among youth, was used to assess polydrug use. Nine items assessed experimentation, years of use, and frequency of use for tobacco/nicotine, alcohol, and marijuana, such as, “Have you ever smoked cigarettes?” In previous studies of African American youth, the ARBA has demonstrated reliability, with alphas ranging from 0.79 to 0.83, and scores associated with mental and substance use disorders (Teplin et al. 2005). A composite score of polydrug use was created based on current recommendations (Kelly et al. 2015). Three subscales were formed by computing the mean of items across substances to reflect experimentation, years of use, and frequency of current use. The mean of the three subscales was computed to create an overall polydrug use score, with higher scores indicating greater polydrug use. In this sample, Cronbach’s *α* was 0.85.

### Statistical Analyses

Preliminary analyses consisted of a) descriptive statistics, b) computing a cumulative risk factor via factor analysis, and c) testing invariance across sex with a measurement model. Main analyses comprised of direct and interaction effects of protective factors using structural equation modeling, along with follow-up post-hoc tests for any significant interactions. Cohen’s *d* effect sizes were also computed using the standardized regression coefficients (standardized beta values). Participants from the same household were clustered to account for non-independent observations (Liu et al. 2015; Liu et al. 2016). All variables were standardized using Aiken and West’s recommended practices (1991) prior to analyses.

Confirmatory factor analysis (CFA) was used to test whether a cumulative risk score was appropriate for the data. In CFA, a latent factor is constructed from measured variables while accounting for correlations among them (Costello and Osborne 2005). A cumulative risk approach was adopted to simplify the statistical models involving multiple risk factors, and to increase generalizability to high-risk environments in which stressors co-exist (Zimmerman et al. 2013). Cumulative risk scores often also account for more variance in outcomes than independently tested risk variables (Luthar 2006). Model fit was evaluated according to standard fit indices: non-significant chi-square, comparative fit index (CFI) greater than 0.95, and root mean square error of approximation (RMSEA) less than 0.06 (Jackson et al. 2009).

Because there may be sex differences in adolescent behavioral problems, measurement invariance tested whether the same psychometric properties of constructs in this study were generalizable across sex. All four levels of measurement invariance (configural, metric, scalar, and strict) were tested, with each level building upon the previous by sequentially adding equality constraints on model parameters to establish invariance across groups. Model fit indices at each level of invariance were used to determine best fit, according to the guidelines above.

Structural equation modeling (SEM) was used to test the associations and interactions among cumulative risk, protective factors, and behavioral health outcomes. SEM allows for the specification and estimation of complex statistical path models, with intervening variables between independent and dependent variables (Merkle et al. 2015). Cumulative risk and the protective factors, including intrapersonal, interpersonal, and neighborhood level factors, were placed into one statistical model to reflect Bronfenbrenner’s (1994) social-ecological model that acknowledges the complex interplay between multiple levels of the environment.

## Results

Statistical analyses were computed using SPSS 22.0 (IBM Corporation, Armonk, NY, United States) and R 3.3.2 (R Core Team, 2016). Structural equation modeling was performed using semTools ( 2016) and lavaan (Rosseel 2012). Analyses were conducted with the full set of 576 individuals with complete data. Subsequently, analyses were re-computed based on the 98.8% of participants who self-identified only as African American. There were no substantive changes in any of the results, so data from the full dataset were reported.

### Confirmatory Factor Analysis

The cumulative risk latent factor, extracted via principal components analysis, had four indicators, exposure to stressors, exposure to violence, racial discrimination, and neighborhood ecology. Kaiser-Meyer-Olkin measure of sampling adequacy was 0.63 and Bartlett’s test for sphericity was significant (χ^2^ (6) = 244.26, *p* < 0.001). One factor was extracted (eigenvalue of 1.81), explaining 45.33% of the variance. All factor loadings were above 0.50. Thus, the cumulative risk factor was retained for use in the structural model.

### Descriptive Statistics

Descriptive statistics and correlations among variables are shown in Table 1. Internalizing and externalizing problems were highly correlated in this sample, as previously reported by our group (Liu et al. 2015). Polydrug use was also correlated with internalizing and externalizing problems. Due to the observed correlations among the behavioral health outcomes, the structural equation model examined all three outcomes in one model to account for covariances.

**Table 1:** Correlations of Protective Factors, Cumulative Risk, and Behavioral Health Outcomes

	1	2	3	4	5	6	7	Mean (*SD*)
Sex	−0.04	0.12**	0.22***	−0.04	0.28***	0.12**	−0.08	
1.Cumulative risk factor	1	−0.13**	−0.22***	−0.14**	0.35***	0.42***	0.23***	
2.Religiosity		1	0.32**	0.05	0.00	−0.06	−0.04	3.56 (1.92)
3.Parental monitoring			1	0.15***	−0.06	−0.19***	−0.19***	11.09 (4.31)
4.Collective efficacy				1	−0.14**	−0.19***	−0.05	2.76 (0.80)
5.Internalizing problems					1	0.63***	0.17***	48.36 (10.17)
6.Externalizing problems						1	0.35***	51.63 (11.45)
7.Polydrug use							1	2.26 (3.62)

***p < 0.001, **p < 0.01, *p < 0.05, two-tailed. Cumulative risk represents latent factor. Internalizing and externalizing problems were analyzed using raw scores. Mean (SD) are shown as T scores for ease of interpretation. Sex coded as 1 = female, 2 = male

Sex differences were observed for all outcome variables. Compared to males, females exhibited higher internalizing problems (*M*_Females_ = 11.05, *SD* = 7.76; *M*_Males_ = 6.94, *SD* = 6.01; *t*(594) = −7.11, *p* < 0.001) and externalizing problems (*M*_Females_ = 12.41, *SD* = 9.19; *M*_Males_ = 10.29, *SD* = 8.15; *t*(594) = −2.93, *p* < 0.01). For polydrug use, there was a non-significant trend whereby males reported slightly higher polydrug use compared to females (*M*_Females_ = 1.97, *SD* = 3.44; *M*_Males_ = 2.47, *SD* = 3.78; *t*(574) = 1.66, *p* = 0.09). Factorial invariance was tested to see whether these sex differences precluded assessing males and females in one statistical model.

### Factorial Invariance

Invariance tests were conducted on a baseline model that included cumulative risk and all outcome measures. Criteria for configural invariance, which carried no equality constraints, was met, indicating that the same items measured cumulative risk across sex (χ^2^ (30) = 47.23, CFI = 0.98, RMSEA = 0.04). Metric invariance, in which factor loadings were constrained to be equal across sex, was also met, indicating that cumulative risk was measured identically and sustained the same meaning to participants across sex (χ^2^ (33) = 53.08, *p* > 0.05, CFI = 0.97, RMSEA = 0.05). Scalar invariance was established by constraining item intercepts and justified mean comparison across sex (χ^2^ (39) = 53.08, *p* > 0.05, CFI = 0.98, RMSEA = 0.04). Finally, criteria for strict invariance, in which factor and error variances were constrained to be equal across sex, was met (χ^2^ (40) = 53.08, p > 0.05, CFI = 0.98, RMSEA = 0.03). Strict invariance established equivalence of residual errors across groups, such that the explained variance for each item was equal across sex, and indicated that the cumulative risk latent construct was comparable across males and females.

### Main and Interactive Effects of Cumulative Risk and Protective Factors

A structural model examined main and interactive effects of cumulative risk and the protective factors with behavioral health outcomes. The model was comprised of the cumulative risk latent factor, three protective factors (observed variables), three behavioral health outcomes (observed variables), and sex. Covariances among internalizing problems, externalizing problems, and polydrug use were modeled, as well as covariances among protective factors. The model showed adequate fit to the data (χ^2^ = 95.49, *p* < 0.05, CFI = 0.92; RMSEA = 0.04).

**Fig. 1:**
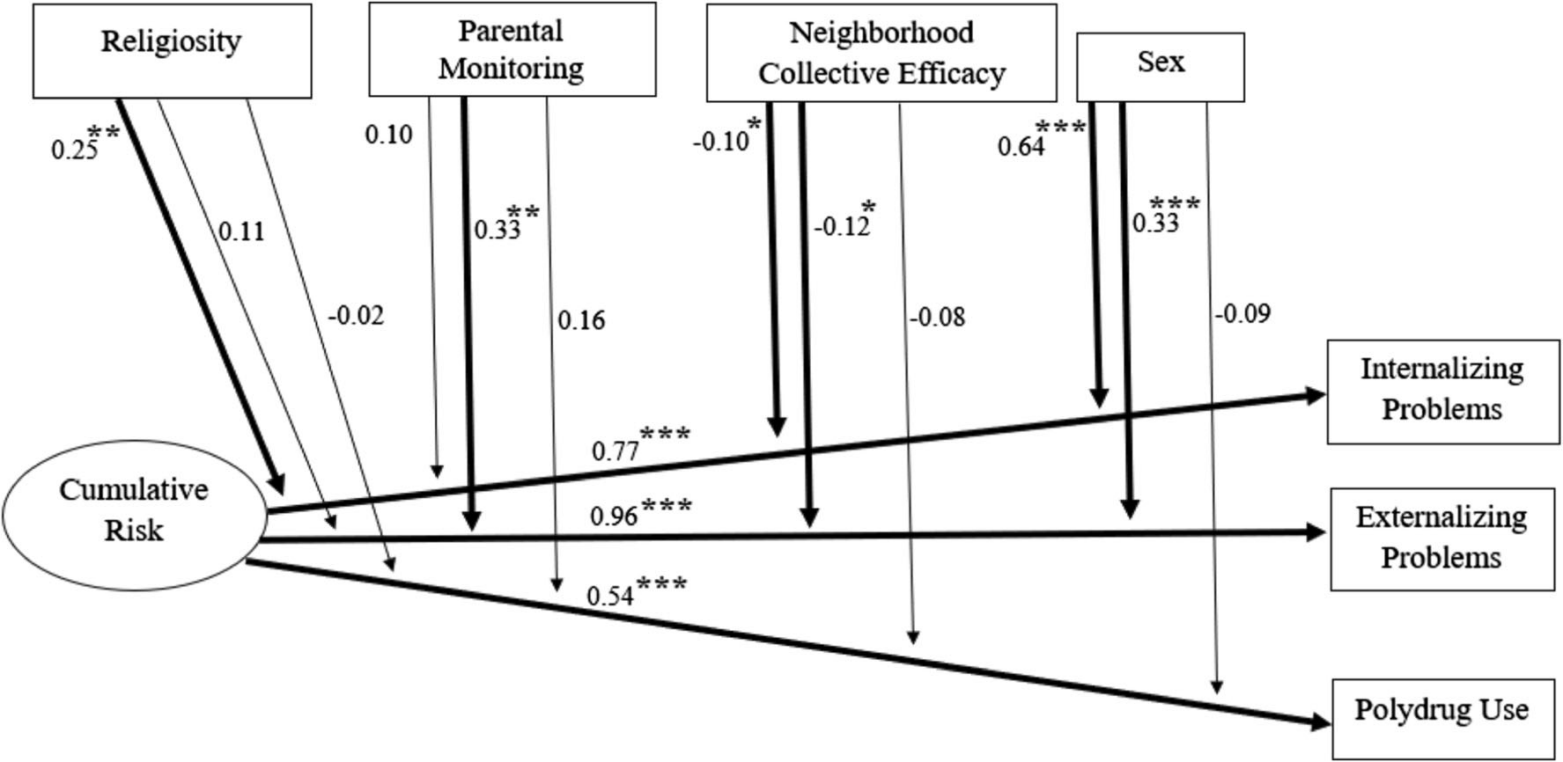
Structural equation model depicting significant associations of cumulative risk, protective factors, and sex with behavioral health outcomes. Child age as a covariate, covariances among protective factors, and covariances among outcome variables were modeled but are not shown. ***p < 0.001, **p < 0.01, *p < 0.05, two-tailed

Results of the SEM model are displayed in Table 2 with significant interactions shown in Fig. 1. Main effects revealed cumulative risk was a significant predictor of outcome variables. Moderate effect sizes were observed, such that higher cumulative risk was associated with higher internalizing problems (Cohen’s *d* = 0.61), externalizing problems (*d* = 0.69), and polydrug use (*d* = 0.51). Protective factors showed negative associations with behavioral health outcomes, with effect sizes somewhat smaller than for cumulative risk. Higher parental monitoring was associated with lower externalizing problems (*d* = 0.38) and polydrug use (*d* = 0.39). Higher collective efficacy was associated with lower internalizing problems (*d* = 0.28) and externalizing problems (*d* = 0.39). Sex showed small to medium effect sizes for internalizing (*d* = 0.59) and externalizing problems (*d* = 0.24), such that females had higher internalizing and externalizing problems than males.

**Table 2:** SEM Model of Two-Way Interactions of Cumulative Risk and Protective Factors on Behavioral Health Outcomes

	Beta	*SE*	Z-value	P(>|z|)
*Internalizing problems*				
Cumulative risk	0.66	0.09	6.95	< 0.001***
Religiosity	0.02	0.04	0.43	0.67
Parental monitoring	−0.06	0.04	−1.50	0.13
Collective efficacy	−0.09	0.04	−2.23	0.03*
Sex	0.66	0.08	8.70	< 0.001***
Cumulative risk*religiosity	0.15	0.05	2.91	< 0.01**
Cumulative risk*parental monitoring	0.11	0.06	1.87	0.06
Cumulative risk*collective efficacy	−0.10	0.04	−2.26	0.02*
*Externalizing problems*				
Cumulative risk	0.78	0.10	7.97	< 0.001***
Religiosity	0.01	0.04	0.24	0.81
Parental monitoring	−0.16	0.04	−3.86	< 0.001***
Collective efficacy	−0.13	0.04	−3.37	< 0.001***
Sex	0.38	0.07	5.15	< 0.001***
Cumulative risk*religiosity	0.06	0.05	1.14	0.26
Cumulative risk*parental monitoring	0.25	0.06	4.32	< 0.001***
Cumulative risk*collective efficacy	−0.12	0.04	−3.00	< 0.01**
*Polydrug use*				
Cumulative risk	0.43	0.09	5.02	< 0.001***
Religiosity	0.05	0.04	1.23	0.22
Parental monitoring	−0.15	0.04	−3.54	< 0.001***
Collective efficacy	−0.04	0.04	−1.01	0.32
Sex	−0.04	0.08	−0.49	0.63
Cumulative risk*religiosity	−0.02	0.05	−0.38	0.70
Cumulative risk*parental monitoring	0.11	0.06	1.84	0.07
Cumulative risk*collective efficacy	−0.08	0.04	−1.83	0.07

***p < 0.001, **p < 0.01, *p < 0.05, two-tailed

Results revealed four significant two-way interactions. Examining internalizing problems, religiosity significantly moderated the association of cumulative risk and internalizing problems (*d* = 0.23). Among adolescents reporting higher religiosity, there was a smaller association of cumulative risk and internalizing problems than adolescents who reported lower religiosity. Further, collective efficacy significantly moderated the association of cumulative risk and internalizing problems (*d* = 0.14). Among adolescents reporting higher collective efficacy, compared to adolescents reporting lower collective efficacy, there was a smaller association of cumulative risk and internalizing problems. The results for externalizing problems showed that parental monitoring moderated the association of cumulative risk and externalizing problems (*d* = 0.26). For adolescents reporting higher parental monitoring, a smaller association of cumulative risk and externalizing problems was observed compared to adolescents reporting lower parental monitoring. Additionally, collective efficacy was a significant moderator of the association of cumulative risk and externalizing problems (*d* = 0.18). Among adolescents who reported greater collective efficacy, there was a smaller association of cumulative risk and externalizing problems. Results for polydrug use showed that none of the protective factors in this study significantly moderated the direct association of cumulative risk and polydrug use.

### Moderating Role of Protective Factors Vary by Sex

Next, three-way interaction terms assessing cumulative risk, each protective factor, and sex were added to the model, while preserving all main and two-way interactions. Results revealed three significant three-way interactions, albeit with small effect sizes. To interpret significant three-way interactions, the sample was split by sex for post-hoc two-way interaction tests.

For internalizing problems, a three-way interaction of cumulative risk, collective efficacy, and sex was observed (*d* = 0.19; *β* = 0.10, *Z*(249) = 2.37, *p* < 0.05). Post-hoc analyses revealed that collective efficacy significantly moderated the association of cumulative risk and internalizing problems for males only (*d* = 0.24; Males: *β* = −0.19, *Z*(229) = −2.92, *p* < 0.01; Females: *β* = 0.003, *Z*(229) = 0.06, *p* > 0.05). For externalizing problems, there was a three-way interaction of cumulative risk, religiosity, and sex (*d* = 0.21; *β* = 0.23, *Z*(249) = 2.56, *p* < 0.01) such that religiosity significantly moderated the association of cumulative risk and externalizing problems among females only (*d* = 0.23; Males: *β* = −0.03, *Z*(229) = −0.46, *p* > 0.05; Females: *β* = 0.34, *Z*(229) = 2.79, *p* < 0.01). For polydrug use, there was a three-way interaction of cumulative risk, parenting monitoring, and sex (*d* = 0.27; *β* = 0.33, *Z*(249) = 3.34, *p* < 0.01) such that parental monitoring moderated the association of cumulative risk and polydrug use for females only (*d* = 0.23; Males: *β* = −0.04, *Z*(229) = −0.66, *p* > 0.05; Females: *β* = 0.38, *Z*(229) = 2.86, *p* < 0.01).

## Discussion

This study investigated associations of cumulative risk with internalizing problems, externalizing problems, and polydrug use among a majority African American sample of adolescents residing in high-risk neighborhoods. As hypothesized, high cumulative risk was associated with greater internalizing problems, externalizing problems, and polydrug use. Moderate effect sizes were observed, demonstrating the predictive power of examining cumulative risk for adolescent behavioral problems. This study also tested adolescents’ own perceptions of intrapersonal, interpersonal, and neighborhood level protective factors, highlighting the multiple environmental levels influencing developmental outcomes (Bronfenbrenner 1994), and demonstrated how neighborhood level factors can shape youth development in promotive ways (Garcia-Coll et al. 1996). Results provided evidence that several protective factors were directly associated with behavioral health outcomes, and in some cases moderated the associations of cumulative risk and behavioral health outcomes. Each of the protective factors will be discussed in turn.

Our observation that religiosity moderated the association of cumulative risk and internalizing problems in both sexes, and externalizing problems among females, adds to the growing body of literature suggesting religiosity may serve as an important protective factor for youth. Although the measure was limited in scope as it only asked youth about religious involvement (participation and importance of religion to the self), the findings are consistent with other studies demonstrating that religious involvement buffers the effects of stress and promotes psychological adjustment among African American adolescents, (Yonker et al. 2012) and is associated with lower delinquency problems, depressive symptoms, drug use, and risky sexual behaviors among youth more broadly (Berg et al. 2009; Mattis and Mattis 2011).

Several models have been posited regarding the mechanisms by which religiosity may serve in a protective role for African American youth. It has been noted that houses of worship have historically served multiple social support functions in African American communities, offering health seminars, individual and group counseling, and youth groups (Belgrave and Allison 2013). It has also been suggested that religious involvement impacts youths’ self-esteem and self-efficacy via prayer or via positive appraisals from peers and adults in the community (George et al. 2002). Our study did not specifically assess the potential mechanisms by which religiosity may serve these buffering effects. Nevertheless, the results add to the literature by demonstrating its protective effects in light of cumulative risk, and by highlighting the potentially broader impact of religiosity for adolescent females’ behavioral health outcomes in the context of cumulative risk.

Our observation that parental monitoring, an interpersonal-level protective factor, was directly associated with externalizing problems was consistent with other cross-sectional and longitudinal research showing that lower parental monitoring is associated with greater delinquency, spending time with deviant peers, and poorer mental health (Goldner et al. 2014). Consistent with our hypotheses, parental monitoring moderated the association of cumulative risk and externalizing problems, such that adolescents who reported higher parental monitoring had a lower association of cumulative risk and externalizing problems compared to adolescents who reported lower parental monitoring. The protective influence of parental monitoring may be related to the normative developmental shift in time spent with peers compared to parents during adolescence (Kerr and Stattin 2000). Parents who engage in close monitoring of youth may lower the likelihood of their children becoming involved in risky peer networks (Rai et al. 2003).

Parental monitoring was also directly associated with polydrug use, consistent with other studies of adolescent substance use (Voisin et al. 2017; Mustanski et al. 2017; Rose et al. 2001). Of interest for the present study was the question of whether parental monitoring buffered the association of cumulative risk and polydrug use. We found partial support for this hypothesis in that higher parental monitoring was linked with an attenuated association between cumulative risk and polydrug use among females only. To date, research on whether parental monitoring effects on African American behavioral health outcomes differ by sex has not been extensively reported, although sex differences in the level of parental monitoring have been observed (Varner and Mandara 2014) and may contribute to such differences. One study of adolescents unselected for race and socioeconomic status has documented that parental monitoring is linked with reduced marijuana and alcohol use and delayed sexual debut specifically among females (Dever et al. 2013). However, that study did not examine youth in poverty separately from middle-class youth. To our knowledge, this study is among the few to report parental monitoring may be a salient protective factor for substance use among adolescent African American females residing in high-poverty neighborhoods.

Results indicated that higher collective efficacy, a neighborhood-level protective factor, was directly associated with lower internalizing and externalizing problems. An adolescent’s neighborhood can exhibit both inhibitory and promotive developmental influences (Garcia-Coll et al. 1996). Neighborhoods may influence development through collective efficacy, due to its influence on the sense of community protection and supervision against these risks (Leventhal and Brooks-Gunn 2000). This study demonstrated that collective efficacy also moderated the association of cumulative risk and internalizing and externalizing problems, in line with prior reports of the protective effects of collective efficacy by our group and others (Liu et al. 2016; Morenoff et al. 2001; Sampson et al. 1997). Together, these findings support the notion that collective efficacy, comprised of social cohesion (e.g., friendliness and mutual trust) and social control (e.g., willingness to intervene in negative events) imparts an important protective mechanism for youth as they spend greater time out of the home and in contact with others in the neighborhood (Frohlich et al. 2001).

This study further provides a more nuanced account of the role of collective efficacy in that our sample was large enough to test whether effects varied by sex. Collective efficacy moderated the association between cumulative risk and externalizing problems for both sexes, but moderated the association between stress and internalizing problems for males only. In a prior study of Chicago youth aged 9–13 years, low collective efficacy exacerbated the link between violence exposure with internalizing and externalizing problems among girls but not boys; high collective efficacy was determined not to have protective effects (Browning et al. 2014). One notable difference between that study and ours was whether collective efficacy was determined through parental vs. adolescent (self) report. Adolescents’ own perceptions of social cohesion and control within their neighborhoods may serve as a buffer against behavioral problems. Little is known about why collective efficacy may buffer risk for internalizing problems specifically among males. One possibility may stem from differences in organization of adolescent relationships, with females spending more time in dyadic relationships and males in larger social groups (Rose and Rudolph 2006). It is possible that collective efficacy may be more protective for males’ coping responses to stress. Replication of this finding is warranted.

This study provided support for direct and buffering effects of protective factors on behavioral health outcomes by emphasizing the importance of investigating multiple contexts of an adolescent’s environment, as posited in social ecological models of development (e.g., Bronfenbrenner 1994; Garcia-Coll et al. 1996). Results may also be interpreted in light of stress-buffering models, which emphasize the protective role of positive social interactions on lowering risk-outcome associations, in that the protective factors examined provided youth opportunities for positive social interactions (La Greca and Harrison 2005). This study highlighted the effects of the exposure to multiple stressors in high-risk environments on youth behavioral problems and sheds new light on the role of protective factors as moderators of cumulative risk.

Direct associations found in this study showed that females in this sample had higher internalizing and externalizing problems than males, whereas previous research has typically reported higher internalizing but lower externalizing problems for females compared to males (Moilanen et al. 2010). This observation may be related to the high comorbidity of internalizing and externalizing problems in our sample. Moreover, there is evidence that symptom profiles of depression may differ among African American compared to White youth. African American youth with depressive symptoms self-report anger and aggression more often than White youth (Anderson and Mayes 2010) which may manifest as a high internalizing and externalizing scores among affected youth when using traditional measures (Liu et al. 2016).

As a whole, the moderating role of sex in this study suggest that the protective factors may have somewhat different effects for males and females. A combination of interpersonal and neighborhood-level protective factors buffered the associations of cumulative risk and behavioral health outcomes among males. A combination of intrapersonal and interpersonal-level protective factors, however, buffered the associations of cumulative risk and behavioral health outcomes among females. As we had a sufficient sample size to consider sex effects within the context of a low-income African American sample, this is the first study, to our knowledge, that has investigated the differential sex effects of these three protective factors in concert. This research has the potential to inform points of potential intervention in community prevention programs.

Results should be interpreted in light of several limitations and considerations. First, the cross-sectional design did not allow for assessment of temporal associations between variables. This limits the ability to draw inferences about the directionality of observed effects. Poor behavioral health outcomes may be associated with environmental stress in a bidirectional manner (Timmermans et al. 2010). Although results of the structural model support the effects of cumulative risk on behavioral health outcomes, longitudinal research is needed to determine the transactional nature of the associations examined in this study. Second, measures in this study were adolescents’ self-reports, which bear the risk of over- or under-reporting. This study, however, utilized audio computerized self-administered interviews, which improves accuracy in self-reports, as interviewers do not need to ask sensitive questions (Morrison-Beedy et al. 2008). Self-reports may also change as a function of participants’ age; however, we controlled for age in primary analyses to minimize potential age-related reporting bias. Third, these findings are based on a sample of predominantly African American adolescents residing in high-poverty urban neighborhoods and may not generalize to African American youth in higher income neighborhoods or other racial groups. African American youth, however, are underrepresented in developmental psychology research and encounter higher risk, compared to some other minority groups, to experience maladaptive physical and mental health outcomes (Mustanski et al. 2013). The cumulative risk approach utilized may aid in generalizability to other high-risk populations where multiple stressors co-exist. Finally, because participants resided in majority African American neighborhoods, their experiences of racial discrimination may differ from those in more racially diverse communities.

Although this study investigated a broad range of protective factors across multiple levels of an adolescent’s environment, it was not an exhaustive assessment of all possible protective factors. Other protective factors such as optimism, self-worth, and belongingness at the intrapersonal level and peer and romantic relationships at the interpersonal level may also buffer the effects of cumulative risk (La Greca and Harrison 2005; Sterrett et al. 2014). Finally, these results should be interpreted in consideration of the fact that this study intentionally focused on cumulative risk instead of individual risk factors. The cumulative risk approach may have yielded different results than examining each risk factor individually. However, a cumulative risk approach was adopted for its high generalizability as it is more representative of adolescents’ actual experiences in high-risk environments (Luthar 2006; Zolkoski and Bullock 2012).

In summary, the present study provided strong evidence that cumulative risk is associated with multiple measures of adverse adolescent outcomes: internalizing problems, externalizing problems, and polydrug use. This study also provided further clarity in terms of which protective factors may buffer cumulative risk effects among predominantly African American youth from high-poverty urban neighborhoods and how these specific protective factors differ by sex. This study highlighted the role of protective factors from multiple contexts of an adolescent’s environment ranging from the intrapersonal to the neighborhood level. Future studies should investigate the mechanisms by which various protective factors might shield against the effects of cumulative risk in high-risk environments. These results speak to the need to consider multiple stressors and multiple levels at which preventive interventions may be beneficial in offsetting risk for poor behavioral health outcomes among vulnerable youth.
